# Living with Keratinocytes

**DOI:** 10.1016/j.stemcr.2018.10.005

**Published:** 2018-11-13

**Authors:** Graziella Pellegrini, Michele De Luca

**Affiliations:** 1 Center for Regenerative Medicine “Stefano Ferrari”, Department of Surgery, Medicine, Dentistry and Morphological Sciences, University of Modena and Reggio Emilia, Modena, Italy; 2 Center for Regenerative Medicine “Stefano Ferrari”, Department of Life Sciences, University of Modena and Reggio Emilia, Modena, Italy

## Abstract

A feature distinguishing human hematopoietic and epithelial stem cells from other equally fascinating stem cells is perhaps their easier translation into a clinical setting. We have devoted nearly our entire scientific career in trying to turn our understanding of epithelial stem cell biology into something that could help people suffering from virtually untreatable diseases of squamous epithelia. We have done that as a team, together with our numerous students, postdocs, technicians and valuable collaborators, clinicians, regulators, and, lately, industrial partners. We had rewarding successes and burning failures, but we always did our best. This award, given by friends and colleagues deserving it more than us, has been the most important recognition of our work. Below, we summarize our story.

## Teaser

In this Perspective, Graziella Pellegrini and Michele De Luca think back over their nearly 30-year joint effort in translating epithelial stem cell biology into regenerative medicine.

## Main Text

A few months ago, we reported on the regeneration of virtually the entire epidermis of Hassan, a Syrian child affected by a severe form of junctional epidermolysis bullosa, by means of autologous, transgenic epidermal cultures (Hirsch et al., 2017). Hassan's prognosis was poor and attempting a compassionate use of combined *ex vivo* cell and gene therapy was the only chance for him to survive. Such therapy was still in the initial phases of clinical trials. While flying back to the Burn Unit in Bochum 10 days after the first transplantation, we and Sergio Bondanza, our historical “cell grower,” could not even talk each other. The appearance of a pink and nice epidermis upon removal of bandages from the grafted area was profoundly moving, perhaps the strongest emotion we had in our entire scientific career, and confirmed how awesome science can be. We understood that Hassan's life could be saved.

**Figure:**
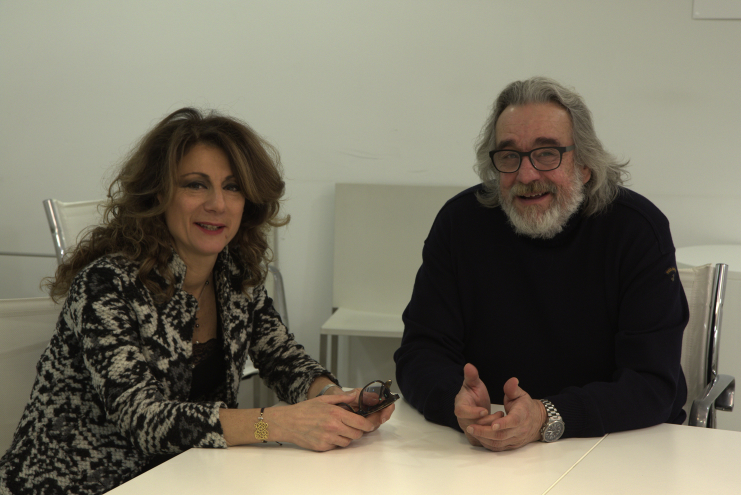
Michele De Luca received his MD at the University of Catania and his specialization in Endocrinology at the University of Rome and is elected member of the European Molecular Biology Organization and the Accademia dei Lincei, Italy. Graziella Pellegrini received her master degrees in Chemistry and Pharmacy at the University of Genova and is founding member of the International Ocular Surface Society. Currently, they are full professors of Biochemistry (M.D.L.) and Cell Biology (G.P.) and coordinators of Cell (G.P.) and Gene (M.D.L.) Therapy at the Center for Regenerative Medicine of the University of Modena and Reggio Emilia. They are co-founders, members of the Board, Scientific Director (M.D.L.), and Director of Research & Development (G.P.) of Holostem Terapie Avanzate S.r.l., Modena, Italy. Michele and Graziella have dedicated their scientific activities to translational medicine and have been a driving force in the development of epithelial stem cell-mediated cell and gene therapy. Following on from early work on the use of human epidermal stem cell cultures in life-saving treatment of massive full-thickness burns and in repigmentation of stable vitiligo and piebaldism by keratinocyte/melanocyte co-culture, they were the first to establish human urethral stem cell culture aimed at urethral regeneration in posterior hypospadias. They have characterized molecular mechanisms regulating long-term proliferative potential, clonal evolution, and self-renewal of epithelial stem cells. These findings led to the use of limbal cultures for corneal regeneration in patients with severe limbal stem cell deficiency due to massive chemical burns of the ocular surface. This treatment allows recovery of normal vision and received marketing authorization from the European Medicine Agency (Holoclar). Michele and Graziella are also pioneering *ex vivo* epidermal stem cell-mediated combined cell and gene therapy for genetic skin diseases. They coordinated the first successful clinical trial to treat junctional epidermolysis bullosa (JEB). They have recently reported life-saving regeneration of the entire, fully functional, epidermis on a 7-year-old child suffering from a devastating, life-threatening form of JEB and showed that the human epidermis is sustained only by a limited number of long-lived stem cells detected as holoclones. They have received numerous awards, including the 2018 International Society for Stem Cell Research (ISSCR) Innovation award (co-awarded), International Prize “Lombardia è ricerca” (co-awarded), Kazemi Award for Research Excellence in Bio-Medicine 2018 (M.D.L.), Eurordis Black Pearl Award 2018 (M.D.L.), Italian Government Award “Le Ragioni della Nuova Politica” 2017 (G.P.), Prix Galien Award Netherlands 2016 and Prix Galien Award United Kingdom 2016 for Holoclar, MIT technology Review Italia “Smart & Disruptive Companies 2015” (co-awarded), ISSCR Public Service Award 2014 (M.D.L.), Biennal Award “A.I.R.C.M.O.” (Italian Association for Research and Treatment of Ocular Pathologies) 2002 (G.P.).

We will tell more about Hassan's story and scientific outcomes, but it is important to stress that this achievement did not come out of the blue. It was the culmination of decades of hard work.

In the mid-1980s, Michele De Luca was a young MD and endocrinologist working as a postdoc on thyroid cells at the NIH in Bethesda. He received a job offer from Ranieri Cancedda, who was heading a tissue engineering laboratory in the newly established National Cancer Institute (IST) in Genova, Italy. Cancedda, knowing Michele's interest in epithelial cells, drew his attention to a paper just published on the *New England Journal of Medicine*, where Howard Green (Harvard Medical School) reported on life-saving regeneration of the epidermis in two severely burned children by means of autologous epidermal cultures (Gallico et al., 1984). Michele couldn't believe it! He read all Green's basic papers behind that extraordinary piece of work, including the first established protocol for primary keratinocyte cultures (Rheinwald and Green, 1975), and tortured Howard Green until he accepted him in his lab. When back in Italy, in 1986, Michele forgot about thyroid and endocrinology and initiated his work on epidermal keratinocytes. Almost at the same time Graziella Pellegrini, after two master degrees in Chemistry and Pharmacy, fascinated by cell and tissue cultures, left her previous research on neuropharmacology to join the laboratory of Ranieri Cancedda. Two years later, Michele and Graziella began working together on human squamous epithelia aimed at regenerative medicine, and they still are.

### Epithelial Stem Cell-Mediated Cell Therapy

We dedicated the first years in Genova to growing epidermal keratinocytes for the treatment of massive, life-threatening full-thickness skin burns. That was quite an exciting period; many patients were saved in collaboration with many Burn Units inside and outside Italy (De Luca et al., 1989, Pellegrini et al., 1999b). However, many patients were lost, due to the infancy of the entire technology (De Luca et al., 2006). We can't forget the first patient in Genova and how challenging it was to simultaneously treat, in collaboration with the Burn Unit in Turin, five young men receiving flame burns on the New Year evening in 1990, as we prepared approximately 500 cultured epidermal grafts in 2 months. We still remember all our traveling, taking the epidermal grafts to the numerous Burn Units and working in close contact with plastic surgeons.

We soon realized that primary epidermal cultures contained melanocytes in close contact with growing keratinocyte colonies. We discovered that human keratinocytes strongly induce melanocyte proliferation, which requires keratinocyte contact and is highly regulated to maintain a physiological keratinocyte/melanocyte ratio. We found that only epidermal keratinocytes have all the information required for the proper physiological organization of melanocytes in the basal layer of the epidermis (De Luca et al., 1988). These findings were the basis for a new clinical application of epidermal cultures, developed initially in Genova and later in Rome, at the Istituto Dermopatico dell’Immacolata (IDI), where we moved in 1996. Patients with stable vitiligo or piebaldism have been successfully treated with cultures optimized to contain a physiological number of melanocytes. The healed epidermis became populated by melanocytes, and stable repigmentation has been attained in those patients (Guerra et al., 2000, Guerra et al., 2004).

Meanwhile, we developed cultures of urethral epithelium, which were successfully applied to regenerate the urethra of children carrying posterior hypospadias, a congenital defect in which the urethra terminates on the ventral surface of the penis (Romagnoli et al., 1990, Romagnoli et al., 1993).

Even though neither Green nor us called cultured keratinocytes stem cells at that time, these human keratinocytes fit the definition of stem cells as we know them today. In those years, by clonal analysis of primary epidermal cultures, Yann Barrandon and Howard Green identified three types of clonogenic keratinocytes, giving rise to holoclones, meroclones, and paraclones (Barrandon and Green, 1987). Over the years, Barrandon's group and we have demonstrated that holoclone-forming cells have all the hallmarks of stem cells and generate meroclones and paraclones that have properties expected of transient amplifying progenitors (Dellambra et al., 2000, Pellegrini et al., 1999a, Pellegrini et al., 1999b, Pellegrini et al., 2001, Rochat et al., 1994, Ronfard et al., 2000).

We applied this notion to a new cell therapy that we initially investigated in Genova at the Center of Advanced Biotechnology (CBA) and further developed first in Rome (IDI), then at the Veneto Eye Bank Foundation in Venice, and finally at the University of Modena and Reggio Emilia (Unimore), where we moved in 2007. We are referring to the use of limbal stem cells for the treatment of severe ocular burns.

Stem cells of the limbus, the narrow zone between the cornea and the bulbar conjunctiva, underpin repair and renewal of the corneal epithelium (Cotsarelis et al., 1989, Schermer et al., 1986). Massive chemical ocular burns may destroy the limbus, causing limbal stem cell deficiency. The cornea acquires an epithelium through the invasion of bulbar conjunctival cells, which leads to neovascularization, chronic inflammation, stromal scarring, corneal opacity, and loss of vision. The only way to prevent this invasion is to restore a normal limbal/corneal epithelium (Kenyon and Tseng, 1989). We initially analyzed the entire human ocular surface and found that holoclones, meroclones, and paraclones constitute also the proliferative compartment of the ocular epithelium. Corneal holoclone-forming cells are strictly segregated in the limbus. Conjunctival holoclones are uniformly distributed in bulbar and forniceal conjunctiva and are bipotent, in that they generate goblet cells at least twice in their life and at rather precise times of their life history, hence through an intrinsic “cell-doubling clock,” whose molecular mechanism is still unclear (Pellegrini et al., 1999a).

An important step toward determining criteria for stem cell content of limbal cultures came from the discovery of p63 (Yang et al., 1998) as a key transcription factor sustaining all squamous epithelia (Mills et al., 1999, Senoo et al., 2007, Yang et al., 1999).

We discovered that ΔNp63α is highly expressed in holoclones, strongly declines during clonal transition from holoclone to meroclone, and is virtually absent from paraclones. In the uninjured surface of the eye, ΔNp63α is present in the limbus but absent from the corneal epithelium. Upon corneal wounding, cells originating from the limbus and containing ΔNp63α migrate progressively through the epithelium of the peripheral and central cornea. In the absence of an attached limbus, no ΔNp63α appears in the corneal epithelium. During this wound-healing process, ΔNp63β and ΔNp63γ appear and correlate with corneal regeneration and differentiation. Accordingly, clonal evolution is marked by a progressive enrichment in ΔNp63β and ΔNp63γ (Di Iorio et al., 2005, Pellegrini et al., 2001). In analyzing the function of p63, we also found that self-renewing human limbal stem cells can be identified by the co-expression of ΔNp63α, C/EBPδ, and Bmi1, rather than ΔNp63α alone. ΔNp63α^+^/C/EBPδ^−^ limbal cells maintain their regenerative capability but have lost their self-renewal. ΔNp63α^+^/C/EBPδ^−^ limbal cells still maintain essential stem cell features, inasmuch as enforced expression of C/EBPδ indefinitely sustains the self-renewal of ΔNp63α^+^ cells but cannot rescue self-renewal in clonogenic ΔNp63α^−^ cells. Thus, ΔNp63α is essential for the proliferative/regenerative capacity of limbal stem cells, while C/EBPδ (and Bmi1) sustain their self-renewal in a ΔNp63α^+^ genetic background (Barbaro et al., 2007).

Altogether, these findings paved the way for the first therapeutic use of autologous limbal cultures for the permanent regeneration of a functional corneal epithelium, leading to recovery of visual acuity not only in patients with unilateral limbal stem cell deficiency but also in those with severe bilateral corneal damage (Pellegrini et al., 1997, Rama et al., 2001, Rama et al., 2010). Indeed, 1–2 mm^2^ of spared limbus in one eye is sufficient to generate limbal cultures able to restore the corneal epithelium of both eyes (Rama et al., 2010). Neither total number of limbal clonogenic cells nor colony size or epithelial cell growth rate could predict clinical outcomes, confirming that the vast majority (∼95%) of clonogenic keratinocytes (meroclones and paraclones) behave as transient progenitors (Pellegrini et al., 2013). This does not mean that the number of clonogenic cells is an irrelevant parameter, in that transient progenitors surrounding epidermal stem cells are instrumental to proper stem cell function, particularly during tissue regeneration (Hsu et al., 2014).

Determination of ΔNp63α abundance in holoclones made it possible to prospectively evaluate the number of stem cells in a limbal culture by computerized biparametric analysis of the cell size and the intensity of p63 staining of single cells (Di Iorio et al., 2006). Strikingly, the clinical success of limbal cultures was strictly related to a precise number of stem cells, defined as p63^bright^ holoclones (Pellegrini et al., 2013, Rama et al., 2010). To date, no other proposed (or supposed) limbal stem cell marker correlates with clinical success of limbal cultures and long-term corneal stability.

In 2015 (almost 20 years after the first proof of principle and hundreds of treated patients), our autologous limbal cultures were complying with the new standards of European regulation and became the first stem cell-based cell therapy to receive marketing authorization from the European Medicine Agency (Holoclar).

But this was quite a bumpy ride. Indeed, in 2007, while dealing with intense clinical application of limbal cultures (in collaboration with over 20 ophthalmology departments) and our first clinical trial on gene therapy of epidermolysis bullosa, the new 1394 Regulation was issued by the European Community, stating that *ex vivo* cell and gene therapy should be considered as advanced therapy medicinal products (ATMPs) and regulated as pharmaceutical drugs. To be approved, in addition to formal clinical trials, ATMPs needed to fulfill good manufacturing practice (GMP), good clinical practice (GCP)—globally referred to as GxPs—requirements, and deal with a variety of documents, including the Common Technical Document. We had to stop all our translational activities and we even thought about giving up: we did not have any GMP-certified laboratory and, more importantly, we realized that the pharmaceutical world with its rules was a different world, requiring a way of thinking and operating quite far from a scientist's mentality. We still remember a long phone call with Howard Green (who wanted to write to the Italian Prime Minister) who, at the end of the phone call, just told us: “you cannot afford to let people down just like that. You have quite a responsibility toward many people with untreatable diseases that you know you can at least try to tackle. You just have to keep going.” He was H (as all his postdocs called him), and we could not say “no” to H as he had been mentoring us for years.

Fortunately, with the help of the “Fondazione Cassa di Risparmio di Modena” (a local Bank Foundation), our University, and Stefano Ferrari (the late Dean of our Faculty), we were able to build the new Center for Regenerative Medicine “Stefano Ferrari” (CMR) containing a large GMP facility. We linked up with Chiesi, a pharmaceutical company in nearby Parma that helped us to create a spin-off biotech, Holostem Terapie Avanzate (HTA). We can't thank enough Andrea and Paolo Chiesi for getting involved in this enterprise. HTA took on the regulatory challenges and made our product compliant with GMP rules. Slowly we came to understand what the European Medicines Agency and our national regulators (AIFA) were afraid of and demanding, and we began planning experiments to give them the reassurances they needed. Overall, however, we lost many years during which also our scientific production decreased substantially, due to the regulatory-type activities we had to perform. Were these requirements appropriate? Yes, because of the improper use of stem cells. While we were working on the construction and certification of the CMR, we were fighting the “Stamina case” in Italy, as worried members of our scientific community (Bianco et al., 2013). The Stamina case has made Italy a battlefield for local and international commercial interests and anti-regulatory lobbies. Italy came close to deregulating mesenchymal stromal cell “therapies” by reclassifying them as transplants, which would have bypassed all the above regulations and opened the way to peddlers of unproven therapies (Bianco et al., 2013). We and other Italian scientists fortunately blocked this. The 2014 Public Service Award, later granted to Paolo Bianco, Elena Cattaneo, and Michele De Luca by the International Society for Stem Cell Research (ISSCR), is a token of the global impact of the issues, as raised by many similar cases around the world.

### Combined *Ex Vivo* Cell and Gene Therapy of Junctional Epidermolysis Bullosa

At the beginning of the 1990s, when still in Genova at CBA, we decided to develop epidermal stem cell-mediated combined *ex vivo* cell and gene therapy. The first successful attempt to introduce and express foreign genes into primary human keratinocytes dates back to 1987 (Morgan et al., 1987). We first demonstrated stable transduction of epidermal stem cells with cDNA carrying β-galactosidase and interleukin-6, the latter secreted in the bloodstream (Mathor et al., 1996). Looking for a skin disease that could be tackled by gene therapy, we came across epidermolysis bullosa (EB). When you know EB kids, they get under your skin. It is really heartbreaking dealing with them. EB is a group of devastating, sometimes early lethal, genetic disorders characterized by structural and mechanical fragility of skin and mucosal membranes, highly impairing the quality of life. There is no cure for EB. The available symptomatic treatments are palliative and can only relieve the ravaging clinical manifestations.

Again, it took many years of investigations before a successful clinical trial was realized. We and others initially discovered that α6β4 integrins mediate adhesion of basal epidermal keratinocytes to the basement membrane at hemidesmosomes (De Luca et al., 1990, Pellegrini et al., 1992, Sonnenberg et al., 1991, Stepp et al., 1990). It has since been uncovered that laminin-5 (now renamed laminin-332) is the primary bridge between hemidesmosomal α6β4 integrins and type VII collagen, the major component of dermal anchoring fibrils (Rousselle et al., 1997). In turn, collagen XVII (also known as bullous pemphigoid antigen 180) links α6β4 integrins to basal keratinocytes intermediate filaments through plectin and BP230 (Borradori et al., 1997, Hopkinson and Jones, 2000), hence completing the adhesion machinery tightening human epidermis to the underlying dermis. To our knowledge, Elaine Fuchs was the first to demonstrate that mutations in genes encoding one component of the above adhesion machinery, namely *KRT5* and *KRT14*, cause EB simplex (EBS), the most common form of EB (Coulombe et al., 1991, Vassar et al., 1991). It was then quite natural to discover that mutations in virtually all genes forming the hemidesmosomal adhesion machinery were responsible for different forms of EB (see Fine et al., 2014 for a review).

There are four major types of EB defined by ultrastructural sites of blister formation, genetics, mode of inheritance, and clinical manifestations. EBS is predominantly due to mutations in keratin 5 and 14 and plectin. Dystrophic EB is due to mutations in *COL7A1*, the gene encoding collagen VII. The absence (or alteration) of anchoring fibrils induces blister formation at the level of the lamina densa and is characterized by severe scarring. Kindler syndrome is due to mutations in the gene encoding kindling-1, a focal contact protein expressed in basal keratinocytes. We decided to tackle generalized junctional EB (JEB). In JEB, blisters and erosions of the skin and mucosa occur within the lamina lucida of the basement membrane in response to minor trauma. Massive chronic skin wounds greatly impair patients’ quality of life, lead to recurrent infections, and predispose patients to skin cancer. JEB is caused by mutations in three genes—*LAMA3*, *LAMB3*, or *LAMC2—*that jointly encode laminin-332 (a heterotrimeric protein, consisting of α3, β3, and γ2 chains) and in genes encoding collagen XVII and α6β4 integrins. More than 40% of patients die before adolescence (Fine et al., 2008).

We first demonstrated genetic and functional correction of *LAMB3*-derived epidermal stem cells *in vitro* (Dellambra et al., 1998). After joining Unimore, we performed a phase I/II clinical trial on *LAMB3*-dependent JEB, which provided compelling evidence that local transplantation of transgenic epidermal cultures, genetically modified by a retroviral vector expressing the full-length *LAMB3* cDNA, can generate a functional epidermis, leading to permanent (follow-up to date being 13 years) correction of JEB skin lesions (De Luca et al., 2006, De Rosa et al., 2014). However, we had to put our trial on hold because of the new regulations issued in 2007. In 2014, we resumed our trial by treating, in collaboration with the EB House and the Dermatology Department in Salzburg, a selected body site of a *LAMB3*-JEB patient, and confirmed the clinical success observed with the first trial (Bauer et al., 2017).

Now we can return to Hassan, who allowed a big leap forward for the development of EB gene therapy. The story of Hassan is touching. He suffers from a severe form of JEB, which could not be controlled in Syria because of the civil war. After a period in Lebanon, his family escaped from that difficult situation and was welcomed in Germany. For a number of reasons, it was too late to control Hassan's disease, which progressively worsened. Due to massive generalized infection from *Staphylococcus aureus* and *Pseudomonas aeruginosa*, Hassan lost most of his skin. In 2015, the child was admitted to the Burn Unit of the Children's Hospital at Ruhr University in Bochum, Germany. Tobias Hirsch, Tobias Rothoeft, Norbert Teig, and the entire clinical staff did an awesome job to keep him alive, but all therapeutic attempts failed and Hassan's prognosis was very poor. Our German colleagues then contacted us for a last experimental attempt. After obtaining authorization from German regulatory authorities, literally the entire CMR in Modena (from University staff to HTA personnel) was concentrated on this enterprise. We generated almost 1 m^2^ of transgenic epidermal cultures and flew to Bochum, where these grafts were applied in collaboration with the German clinicians, with two big surgical procedures in 2 months.

Such combined *ex vivo* cell and gene therapy has proved to be life saving, inasmuch as it was able to regenerate virtually the entire epidermis of Hassan (Hirsch et al., 2017). At the last follow-up, almost 3 years after grafting (i.e., over 30 cycles of epidermal renewal), Hassan's transgenic epidermis expressed normal levels of laminin-332, had normal thickness and continuity of the basement membrane, and normal morphology of hemidesmosomes (Hirsch et al., 2017). Hassan's transgenic epidermis is robust and resistant to mechanical stress and does not develop blisters or erosions. Hassan is currently leading a normal social life. He is exercising, playing soccer, and injuring himself with normal wound healing. Currently we and others have initiated several *ex vivo* gene therapy clinical trials trying to tackle different forms of EB (Siprashvili et al., 2016).

Genome-wide integration profile of his transgenic epidermis confirmed the absence of clonal selection both *in vitro* and *in vivo*. Indeed, Hassan received ∼4 × 10^8^ transgenic clonogenic keratinocytes and did not manifest tumor development or other related adverse events (Hirsch et al., 2017). The transgenic nature of the regenerated epidermis, the notion that proviral integrations provide a clonal genetic mark, and the possibility to analyze clonogenic keratinocytes at clonal level helped to solve a controversy in the field. By clonal tracing experiments, we formally proved that human epidermis is sustained only by a limited number of long-lived stem cells detected as holoclones that give rise to pools of short-lived progenitors (meroclones and paraclones), which persist for various periods of time, replenish differentiated cells, and make short-term contributions to wound healing (Hirsch et al., 2017). Until then, this notion was sustained only by indirect, though compelling, evidence (Pellegrini et al., 1999b, Ronfard et al., 2000), including the observation that clinical success of limbal cultures requires a defined percentage of p63^bright^ holoclones (Pellegrini et al., 2013, Rama et al., 2010). This last notion is highly relevant, in that it shows that the essential feature of any epithelial grafts is the presence of holoclone-forming cells, which are instrumental for long-term epidermal renewal. After this criterion is met, success depends exclusively on clinics. Most likely, the loss of holoclone-forming cells, supposedly due to improper culture conditions, accounts for some failures of long-term epithelial regeneration by means of epidermal and corneal cultures (De Luca et al., 2006).

This is the essence of our scientific career, which led us to be chosen to receive the 2018 ISSCR Award for Innovation. There is no greater honor for us than to be recognized by top scientists working in our same field, scientists for whom we have enormous respect and esteem; scientists who stated that this prestigious award was motivated by our perseverance in trying to combine basic science and highly controlled translation medicine to help people with virtually untreatable disease. While struggling with regulations and climbing the steep, long, and winding road toward clinical translation of such advanced therapies, we also had to fight quacks selling unproven “stem” cell therapies. As our colleagues told us while congratulating us for this award, we also hope that our work could provide young scientists with a blueprint that can lead them to successfully develop other fields of regenerative medicine. The past decade has been marked by other stem cell-based therapies producing remarkable clinical results on incurable diseases, such as hematopoietic stem cell gene therapy of immunodeficiencies, Wiskott-Aldrich syndrome, or metachromatic leukodystrophy (Aiuti et al., 2009, Aiuti et al., 2013, Biffi et al., 2013, Cavazzana-Calvo et al., 2000). In 2016, gene therapy for adenosine deaminase severe combined immunodeficiency became the first stem cell-based gene therapy to receive marketing authorization from the European Medicine Agency (Strimvelis). But we are also witnessing an exponential growth of improper use of ATMPs that, in most cases, lacks a scientific rationale and whose preclinical safety and efficacy are unclear and inconclusive. As expected, they were not efficacious and often detrimental for the patients and the whole field of stem cell-based regenerative medicine (Bianco et al., 2013).

We could not imagine focusing on any science not involving our beloved keratinocytes and their use in regenerative medicine. Thus, our heartfelt thanks go to our Chiesi/Holostem industrial partners, who are trying to turn our science into real therapies for all people suffering from such severe diseases, and to the many clinicians collaborating in the development of different fields of regenerative medicine. Our last thought, however, goes to the numerous BSc and PhD students, postdocs, technicians, and staff we had the honor to mentor in these years. They were and are our victims, since they too had to love keratinocytes before being allowed to work with them. They are too many to be named, but without them all this work would not have been possible. It would be hypocritical to deny that some of them have been and are special.
